# Microbial Products Induce Claudin-2 to Compromise Gut Epithelial Barrier Function

**DOI:** 10.1371/journal.pone.0068547

**Published:** 2013-08-21

**Authors:** Xiaoyu Liu, Gui Yang, Xiao-Rui Geng, Yanjuan Cao, Na Li, Li Ma, Si Chen, Ping-Chang Yang, Zhigang Liu

**Affiliations:** 1 State Key Laboratory of Respiratory Disease for Allergy at Shenzhen University, Shenzhen Key Laboratory of Allergy & Immunology, Shenzhen University School of Medicine, Shenzhen, China; 2 Longgang Central Hospital, ENT Hospital, Shenzhen ENT Institute, Shenzhen, China; University Hospital Hamburg-Eppendorf, Germany

## Abstract

The epithelial barrier dysfunction is an important pathogenic feature in a number of diseases. The underlying mechanism is to be further investigated. The present study aims to investigate the role of tight junction protein claudin-2 (Cldn2) in the compromising epithelial barrier function. In this study, the expression of Cldn2 in the epithelial layer of mice and patients with food allergy was observed by immunohistochemistry. The induction of Cldn2 was carried out with a cell culture model. The Cldn2-facilitated antigen internalization was observed by confocal microscopy. The epithelial barrier function in the gut epithelial monolayer was assessed by recording the transepithelial resistance and assessing the permeability to a macromolecular tracer. The results showed that the positive immune staining of Cldn2 was observed in the epithelial layer of the small intestine that was weakly stained in naïve control mice, and strongly stained in sensitized mice as well as patients with food allergy. Exposure to cholera toxin or Staphylococcal enterotoxin B induced the expression of Cldn2 in HT-29 or T84 cells. Cldn2 could bind protein antigen to form complexes to facilitate the antigen transport across the epithelial barrier. Blocking Cldn2 prevented the allergen-related hypersensitivity the intestine. We conclude that the tight junction protein Cldn2 is involved in the epithelial barrier dysfunction.

## Introduction

An epithelial layer covers on surface of the intestine [1]. The epithelial cell bodies and the tight junction complexes between adjacent epithelial cells form the epithelial barrier, which seals the deep tissue from the external environment. An integrated epithelial barrier is a critical structure involving in maintaining the homeostasis in the body. The dysfunction of epithelial barrier is associated with a number of diseases, such as allergic diseases [2]–[4] and chronic inflammation [5], [6]. Yet, factors causing the epithelial barrier dysfunction are not fully understood yet.

The tight junction complex is composed of several molecules such as claudins, occludins and junction adhesion molecules [7]. The claudins are a large family; 24 members of claudins have been described [7]. Published data indicate that not every member of claudins contributes to the integrity of the tight junctions; some of them have a negative effect on the epithelial barrier function. Claudin-2 (Cldn2) is found to form channels that facilitate the ion secretion by epithelial cells [8]. Cldn1, Cldn6 and Cldn9 are proposed to be the entry cofactors for hepatitis C virus [9]. Previous reports indicate that the elevation of ion secretion can be accompanied with the elevation of macromolecular protein transepithelial transport [4], [10], [11], in which the mechanism remains to be further elucidated. Besides facilitating the ion secretion, whether Cldn2 also facilitates the protein antigen transepithelial transport is unclear.

In the present study, we screened the 24 subtypes of Cldns in the small intestine of mice with allergic disorders and found that Cldn2 was uniquely increased. We postulate that Cldn2 plays a role in compromising the epithelial barrier function in the intestine, which may be involved in the development of intestinal allergy. Since microbial adjuvants can facilitate the establishment of food allergy animal models [12]–[14], intestinal epithelial barrier dysfunction is one of the features of the induced food allergy model [4], we treated the intestinal epithelial cells with Staphylococcal enterotoxin B (SEB) or cholera toxin (CT), which are powerful adjuvants using in establishment of intestinal allergic animal models. The results showed that exposure to SEB or CT promoted the expression of Cldn2 in the intestinal epithelial cells; Cldn2 facilitated absorption of protein antigens; blocking Cldn2 prevented the development of food allergy in a murine model.

## Materials and Methods

The experimental procedures of the animal model were approved by the Animal Care Committee at Shenzhen University.

### Reagents

Anti-Cldns, anti-horseradish peroxidase (HRP), Cldn2 short hairpin (sh) RNA and control shRNA were purchased from Santa Cruz Biotech (Santa Cruz, CA). Immunoprecipitation kit, cholera toxin, Staphylacoccal enterotoxin B (SEB) and HRP (type II) were purchased from Sigma Aldrich (Oakville, ON, Canada). Reagents of quantitative real time RT-PCR (qRT-PCR) were purchased from Bio-Rad (Mississauga, ON, Canada). The ELISA kits of IL-4, IL-5 and IL-13 were purchased from R&D Systems (Burlington, ON, Canada).). The IgE ELISA kit was purchased from AbD Serotec (Raleigh, NC). Polyclonal anti-Cldn2 antibody and gold conjugated antibody were purchased from Abcam (Cambridge, MA). Endotoxin levels in all reagents were detected using the Limulus assay (Limulus amebocyte lysate QCL 1000, Bio Whittaker, Walkersville, MD, USA). The reagents used in this study contained <0.2U endotoxin/10 µg reagents.

### Collection and processing of mouse intestinal epithelial tissue

The jejunal segments were excised from mice at sacrifice. The epithelial tissue was collected by gently scrapping on the surface of the inner surface of the jejunal segments with a glass slide. The total RNA and proteins were extracted respectively following the standard operating procedures in our laboratory that were also described in the Information S1.

### Co-localization and immunofluorescence assays

Following our published procedures [15] with minor modification, HT-29 or T84 cells were collected from the culture and washed with cold phosphate-buffered saline (PBS) and fixed with cold methanol on ice for 30 min. The fixative was aspirated and the cells were washed with cold PBS 3 times and blocked with 100 µl blocking buffer (1% bovine serum albumin and 0.1% Triton-X 100 in PBS; using the Triton-X100 to promote the penetrating ability of reagents) for 30 minutes at room temperature. The cells were incubated with primary antibodies (rabbit anti-Cldn2, 1∶200; mouse anti-HRP, 1∶300) for one hour at room temperature. Cells were washed with cold PBS 3 times and incubated with the fluorescence-conjugated secondary antibodies (FITC-anti-rabbit IgG, 1∶300; Cy5-anti-mouse IgG Fab, 1∶300) for one hour. After washing with PBS for 3 times, the cells were smeared on slides, mounted with cover slips, and observed with a confocal microscope (LSM510).

### Immunoprecipitation

The HT-29 and T84 cells were washed three times with cold PBS and lysed with lysis buffer (150 mM NaCl, 20 mM Tris–HCl pH 7.4, 5 mM EDTA, 1% NP-40, 1% Na-deoxycholate, 0.1% SDS, 1 mM PMSF, 20 µg/ml Leupeptin, 20 µg/ml Aprotinin, 3 µg/ml Pepstatin A). After centrifugation at 15,000 RPM for 15 min, the supernatant was collected. The proteins (500 µg) were pre-cleared with 20 µl of protein G agarose/sepharose bead slurry (50%), and then 1 µg of anti-cldn2 antibodys was added and rotated at 4°C overnight. The protein G Sepharose (20 ml) was added to the mixture and mixed for 1 h at 4°C. The Protein G gel was washed 3 times with lysis buffer, resuspended with 20 µl of SDS sample buffer, incubated at 100°C for 5 min and subjected to SDS-PAGE. The remained procedures were the same as Western blotting.

Some experimental procedures were presented in Information S1.

## Results

### Cldn2 is increased in the intestinal epithelia of mice with antigen-specific hypersensitivity

**Figure 1:**
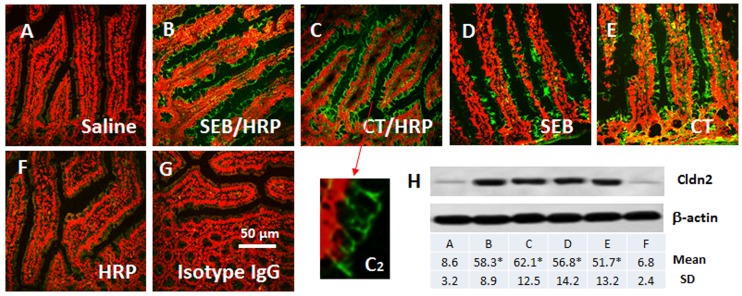
Expression of Cldn2 in the mouse intestinal epithelium. Grouped mice were sensitized to HRP or only treated with adjuvant SEB or CT. A–G, representative confocal images show the expression of Cldn2 (in green) on the epithelia. Panel C2 is an enlarged image spot in panel C (pointed by an arrow). The red color is the nucli that was stained with propidium iodide. H, the immune blots show the Cldn2 protein (22 kDa) in the mouse jejunal epithelia. The table below indicates the summarized integrated density of the immune blots. *, p<0.01, compared with the saline group. Each group consisted of 6 mice. Samples from each mouse were processed separately.

With an intestinal allergy mouse model, we examined the expression of Cldns in the jejunal mucosa. The food allergy parameters of the mice were presented in the (Fig. S1, S2, S3, S4 in Information S1). To assess the expression of Cldns in the epithelium, we collected the mucosal tissue from the mouse jejunum upon sacrifice. The expressions of the 24 Cldns were assessed by qRT-PCR. As shown in Table 1, the Cldn2 mRNA levels were markedly increased (p<0.01); the Cldn6, 9, 15, 18 and 22 mRNA levels were slightly increased (p>0.05) while the mRNA levels in the rest eighteen Cldns were slightly decreased (p>0.05). The results imply that Cldn2 may be an important factor in the food allergy related intestinal hypersensitivity. To confirm the results, we stained the jejunal sections with anti-Cldn2 antibody. The results showed that the expression of Cldn2 could be detected in the epithelial cells of mice treated with saline (Fig. 1A), which was significantly increased in sensitized mice (Fig. 1B, 1C, 1C2). Considering the sensitized mice were treated with both adjuvants (SEB or CT) and allergen (HRP), in separate experiments, we treated mice with either adjuvant alone, or allergen alone. The mice treated with adjuvants also showed positive staining of Cldn2 (Fig. 1D, 1E), but not in those treated with allergen alone (Fig. 1F). The staining was further confirmed by Western blotting with samples collected from the jejunal epithelium (Fig. 1H).

**Table 1:** Cldn primers and qRT-PCR results (%β-actin).

Cldns	Primers		NCBI#	Results	
	Forward	Reverse		Mean	SD
Cldn1	ttttcccgatgacctttctg	agtttgcaggatctgggatg	NM_016674	−2.8	0.6
Cldn2	aaggtgctgctgagggtaga	ttgagcattcaaagcacagg	NM_016675	780.5*	65.2
Cldn3	gcacccaccaagatcctcta	agcctgtctgtcctcttcca	NM_009902	−1.8	0.4
Cldn4	acaggtcctgggaatctcct	cactgcatctgacctgtgct	NM_009903	−2.6	0.5
Cldn5	gctctcagagtccgttgacc	ctgccctttcaggttagcag	NM_013805	−3.3	0.8
Cldn6	tgagtccaagctccacctct	cttgggactgggacaggata	NM_018777	1.9	0.5
Cldn7	agcatgttcctggattggtc	cctggacaggagcaagagag	NM_001193619	−2.2	0.5
Cldn8	tcccaaggcgtacagatttc	cactctccactgaggcatga	NM_018778	−3.6	1.2
Cldn9	taatgggtgaaggcttccag	gctcttggcctgaatcactc	NM_020293	18.2	6.8
Cldn10	gtctgtgattaccgccacct	gccaagcaagcaattttagc	NM_001160098	−2.9	1.1
Cldn11	accacttcttggctgcctta	acccaagtcagcaatgttcc	NM_008770	−7.7	2.9
Cldn12	ttgaatgtgagacggtgcat	taaacatgaggcagcacagc	NM_001193659	−6.5	2.6
Cldn13	acttgctggagctcgacatt	ctcttgtttgctgacgacca	NM_020504	−1.2	0.4
Cldn14	aatgaggcaaagccagaaga	aggaagcctaggagctggac	NM_001165926	−1.6	0.5
Cldn15	ctctacttgggctggagtgc	caggccagagcttcctacac	NM_021719	21.3	7.2
Cldn16	tttacgtcgaacgctcctct	tgaatagggcttcctcatgg	NM_053241	−3.3	1.2
Cldn17	gccaaggatgagaatggaaa	tccatctgagggaactttgg	NM_019753	−4.2	1.6
Cldn18	gagtgccgaggctacttcac	agatgccggagatgatgaac	NM_001194921	8.6	2.8
Cldn19	tcctcttggcaggtctctgt	gtgcagcagagaaaggaacc	NM_153105	−4.4	1.5
Cldn20	gtgtctggggtgtctggagt	gtacaaatcccccaagcaga	NM_001101560	−2.8	1.2
Cldn22	agggtcttgatgtccctgtg	tggtctcatcccagaactcc	NM_029383	9.5	4.2
Cldn23	ctccctgcttcagtctccag	ggtatctcgggaacaggaca	NM_027998	−1.2	0.5
Cldn26	ctcagtggatggcaaccttt	tcaggtgataagggctccac	NM_029070	−3.3	1.4
Cldn27	tccaacctagggagctgaga	tgggaaagggaacaaaactg	NM_001085535	−5.8	2.3

* , p<0.01, compared with mice treated with saline. The data represent 6 experiments.

### Exposure to microbial products, CT or SEB, increases the expression of Cldn2 in intestinal epithelial cells and compromise the epithelial barrier function

**Figure 2:**
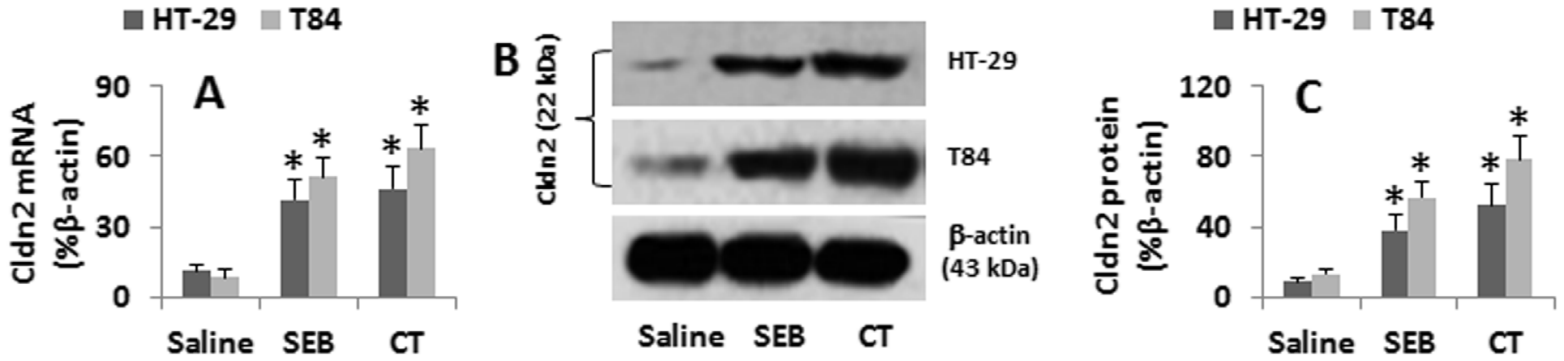
CT and SEB up-regulates Cldn2 expression in gut epithelial cells. A, HT-29 and T84 cells were cultured in the presence of SEB or CT at graded doses for 48 h. B, HT-29 cells were cultured in the presence of CT (500 ng/ml) for 48 h. The cellular extracts were analyzed by qRT-PCR and Western blotting. The bars in A indicate the levels of Cldn2 mRNA. B, the cellular proteins were extracted from the cells and analyzed by Western blotting. The immune blots indicate the levels of Cldn2 protein. C, the bars show the summarized integrated density of the immune blots in B. Another piece of membrane was stained with isotype IgG, no staining was resulted (data not shown). The data in bar graphs were presented as mean ± SD. *, p<0.01, compared with the group saline The data represent 6 separate experiments.

Although the data in Fig. 1 imply that the elevated Cldn2 levels in the jejunal epithelia were related to the treatment with CT or SEB, the results are still somewhat indirect. Thus, we treated HT-29 or T84 cells with CT or SEB in the culture for 48 h. As shown by qRT-PCR and Western blotting, the cells treated with saline showed the baseline Cldn2 expression that was markedly increased by the exposure to SEB or CT (Fig. 2). The data confirm that SEB or CT can up-regulate the expression of Cldn2 in HT-29 and T84 cells.

**Figure 3:**
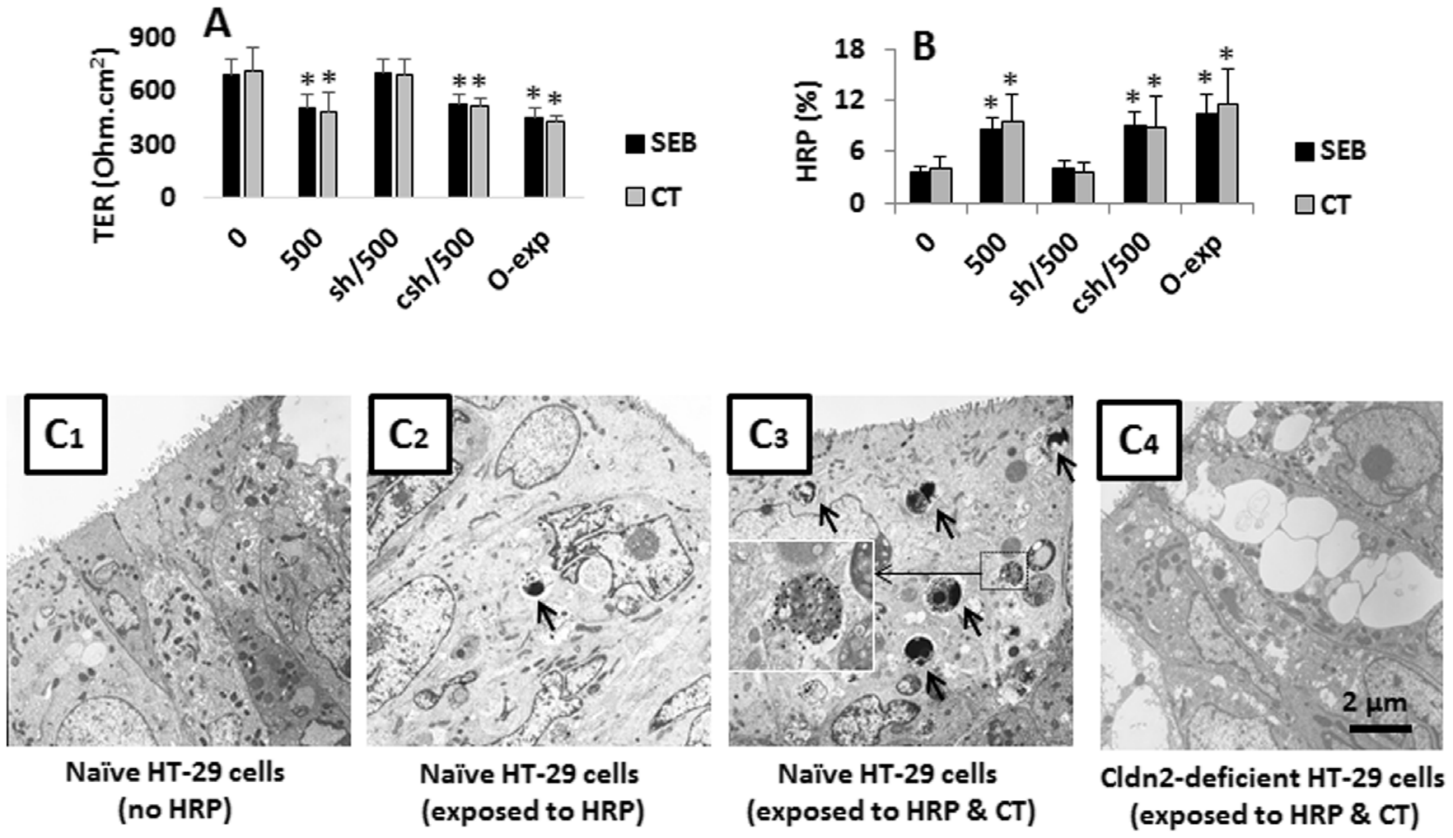
Assessment of gut epithelial monolayer barrier function. Confluent HT-29 monolayers were exposed to SEB or CT (500 ng/ml) in the culture. HRP (20 ng/ml) was added to the apical chamber of transwells. A, the bars indicate the TER of HT-29 monolayers recorded 48 h later. B, the permeability of the epithelial monolayer was carried out in the period of 46 h and 48 h; the bars indicate the HRP levels in the basal chambers of transwells (presented as the percentage of HRP amount added to apical chambers). The data were presented as mean ± SD. *, p<0.01, compared with the group “0”. The X axes of A and B present the treatment of the monolayers; the numbers indicate the concentrations of SEB or CT. sh: HT-29 cells were treated with Cldn2 shRNA. csh: Cells were treated with control shRNA. C, representative electron microscopical images show the uptake of HRP by HT-29 monolayers. Treatments were denoted below each image. The data represent 3 separate experiments.

Based on the results in Fig. 1 and Fig. 2 that the Cldn2 was significantly increased in the gut epithelia of mice after the exposure to SEB or CT, we inferred that the elevation of Cldn2 expression might also induce the epithelial barrier dysfunction. To test the hypothesis, using the same cell culture model in Fig. 2, we assessed the HT-29 monolayer barrier function. The results showed that the TER dropped significantly and the HRP increased markedly as compared with controls, which did not occur in the Cldn2-deficient HT-29 monolayers (Fig. 3A–B; Fig. S5 in Information S1). Using HRP as a tracer, we observed a marked increase of the epithelial permeability of the HT-29 monolayers after exposure to SEB or CT for 48 h, which could be mimicked by the over expression of Cldn2 (Fig. 3A–B; Fig. S6 in Information S1). The results indicate that exposure to SEB or CT can induce the HT-29 monolayer barrier dysfunction, which can be inhibited by knocking down the Cldn2 gene. The results were also reproduced in T84 monolayers (data not shown).

To determine if the HRP transport to the basal chambers of Transwells was also via the intracellular pathway, we examined the HT-29 monolayers by electron microscopy. The HRP endosomes were observed in the cytoplasm of HT-29 cells (Fig. 3C) that were much more in CT-treated group than the medium group. Using the gold particle-conjugated antibody staining, the gold particles were localized on the HRP endosomes (see the insert in Fig. 3C3). The results of Fig. 3C were reproduced in T84 cells or using SEB as the stimulator (data not shown). The data indicate that the macromolecular proteins can be internalized into HT-29 cells after the exposure to CT or SEB in the culture. The HRP endosomes was barely observed in Cldn2-deficient HT-29 (Fig. 3C4) or T84 cells (not shown).

### Cldn2 facilitates the transport of protein antigens across the cell membrane of HT-29 cells

**Figure 4:**
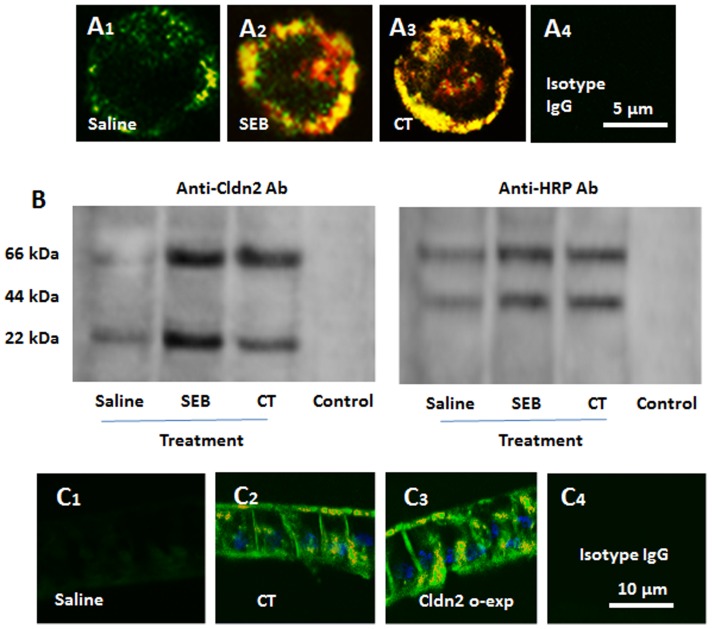
Cldn2 facilitates the internalization of antigen in HT-29 cells. HT-29 cells were cultured in the inserts of Transwells in the presence of CT or SEB (500 ng/ml) or saline for 48 h and then, exposed to HRP in the culture. The cells were removed using tripsin-EDTA from the supporting filter at 30 min later. A, the represent confocal images show positive staining of Cldn2 (in green) and HRP (in red) in the cells. The yellow color is the merged color of red and green. A4 is an isotype IgG control. B, proteins were extracted from the cells and processed by immune precipitation assay (IP). The immune blots indicate an immune complex of Cldn2 and HRP in the extracts (66 kDa) in addition the blots of HRP (44 kDa) and Cldn2 (22 kDa) were localized at their own sites respectively. The protein samples added to the control lane were precipited by isotype IgG. C, HT-29 monolayers were processed and observed by confocal microscopy. The representative images indicate the expression of Cldn2 (in green) and the colocalization (in yellow) of Cldn2 and HRP (in red). The blue staining indicates the staining of the nucli (stained with DAPI). The treatments were denoted in individual images. C4 is an isotype control. Cldn2 o-exp: Cldn2 over-expression. The data represent 3 separate experiments.

Based on the results that exposure to CT or SEB increased the expression of Cldn2 in the intestinal epithelial cells (Fig. 1), the epithelial barrier permeability and internalizes the protein antigens (Fig. 3), we inferred that Cldn2 may facilitate the transport of antigen across the intestinal epithelial barrier. To test the hypothesis, we observed if Cldn2 could bind protein antigens to form complexes. By confocal microscopy, we observed the colocalization of both Cldn2 and HRP on the surface as well as in the cytoplasm of HT-29 cells (Fig. 4A). By immunoprecipitation assay, a complex of Cldn2/HRP was detected in the extracts of the HT-29 cells after exposing to CT and HRP (Fig. 4B). We also obtained similar results in the experiments with T84 cells (data not shown). The expression of Cldn2 and colocalization of Cldn2 and HRP were confirmed with HT-29 monolayers that were treated in the same way of panel A (Fig. 4C). Over-expression of Cldn2 (Fig. S6 in Information S1) also markedly increased the uptake of HRP (Fig. 4C). The results indicate that Cldn2 forms a complex with protein antigens to facilitate the internalization of antigen into gut epithelial cells.

### Inhibition of Cldn2 prevents allergen-related hypersensitivity in the intestine

To evaluate the role of Cldn2 in the induction of intestinal hypersensitivity, we treated mice with CT and HRP (or SEB and HRP; data not shown) with or without the pretreatment with polyclonal anti-Cldn2 antibody. The results showed that an intestinal hypersensitivity mouse model was successfully established manifesting high levels of serum HRP-specific IgE and histamine (Fig. S2 in Information S1), epithelial barrier hyperpermeability to the macromolecular protein HRP (Fig. 3), and the HRP-specific Th2 responses in the intestine (Fig. S3, Fig. S4 in Information S1). The aberrant Th2 responses in the intestine were abrogated by pretreatment with anti-Cldn2 polyclonal antibody (Fig. 2, Fig. S1, S2, S3, S4 in Information S1).

### Expression of Cldn2 is increased in the intestinal epithelium of patients with food allergy

**Figure 5:**
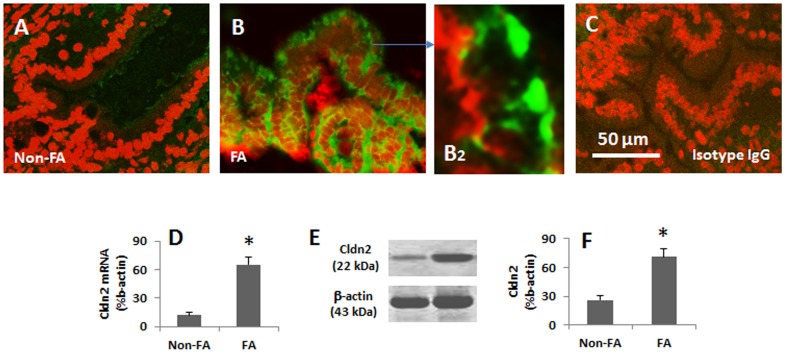
Expression of Cldn2 is increased in gut epithelia of patients with food allergy (FA). The duodenal biopsies were obtained from 6 patients with food allergy and 6 patients with duodenal peptic ulcers (no FA) and processed for immunohistochemistry. A–C, representative confocal images show the Cldn2 positive staining (in green). C is an isotype control. D, the bars indicate the mRNA levels of Cldn2 in the biopsies that were analyzed by qRT-PCR. E, the immune blots indicate the protein levels of Cldn2 in the biopsies that were analyzed by Western blotting. The β-actin blots were used as an internal control. F, the bars indicate the summarized integrated density of the immune blots in E. The samples from patients were analyzed individually. The data represent 6 separate experiments.

Finally we observed the expression of Cldn2 in the human intestinal epithelium of patients with food allergy. The diagnosis of food allergy was presented in Information S1. The demographic data of the patients were presented in Table S1 in Information S1. Duodenal biopsies were obtained from each patient. As shown by confocal microscopy, the weak staining of Cldn2 was detected in the specimens from non-food allergy patients while the strong positive staining was observed in the specimens from patients with food allergy. The results were further confirmed by the data of qRT-PCR and Western blotting (Fig. 5).

## Discussion

A number of factors are associated with the epithelial barrier dysfunction, such as microbial infection [16], neural factors [17], chronic psychological stress [4] and exposure to proinflammatory cytokines [18]. The present study has expanded our knowledge by revealing that Cldn2, one of the components of tight junction proteins, is also an important factor in the induction of epithelial barrier dysfunction. We observed that strong Cldn2 expression in the intestine of mice with intestinal antigen-specific hypersensitivity and in patients with food allergy. Cldn2 could bind protein antigen to form complexes to facilitate the internalization of the antigens in epithelial cells. Blocking Cldn2 abrogated the microbial product-induced epithelial barrier dysfunction and inhibits the development of intestinal allergy.

Cldn2 is one of the recognized tight junction proteins [7]. Besides expressing in the tight junctions, Cldn2 is also expressed at the cilium [19]. A similar observation was reported by Denizot et al that the expression of the pore-forming tight junction protein Cldn2 on the colon epithelial cells in 49% of a group of patients with active Crohn disease [20]. Our results show that in addition to the tight junction and the cilium, Cldn2 is also localized on the surface of the epithelium of the small intestine with allergy. After treating mice with our established protocol to sensitize to a specific antigen, the expression of Cldn2 was significantly increased in the epithelial cells of the small intestine. Since the reagents using in the sensitization contained CT or SEB, exposing to CT or SEB also induced the expression of Cldn2 in HT-29 and T84 cells, we propose that the microbial product CT or SEB can induce the expression of Cldn2 in the epithelial cells of the intestine. The results are in line with a recent study that the exposure to *E. coli* can markedly increase the expression of Cldn2 in the intestine [20]. Some allergens such as the Der P 2 from house dust mite also can up-regulate the expression of Cldn2 in the airway epithelial cells via enhancing the phosphorylation of glycogen synthase kinase-3b and its potential upstream regulator Akt [21]. Increases in the phosphorylation of p38 kinases were observed in the CT-induced Cldn2 expression in the present experiments (data not shown). Others also indicate that the IL-6 induced Cldn2 expression is MEK/ERK and PI3K-dependent [8]. Intestinal epithelial cells express MHC II [22]; CT and SEB can bind MHC II and activates the phosphorylation of p38 kinase [23] and further promotes the expression of Cldn2 as observed in the present study.

The major function of most tight junction proteins is to maintain the integrity of the epithelial barrier. However, some of the tight junction proteins show negative effects on the barrier integrity. Cldn2 can form channels in the tight junction to increase the permeability of the epithelial barrier via increasing the ion secretion [8]. Investigators have also noted that Cldn1, Cldn6 and Cldn9 are entry cofactors for hepatitis C virus [9]. Our results show that Cldn2 down regulates the TER and increases the transcellular permeability to macromolecular proteins in HT-29 monolayers, which is in line with others' observations that Cldn2 facilitates the bacterial translocation in the intestine [20].

One of the functions of the epithelial barrier is to prevent the macromolecular antigens and other noxious substances from being absorbed. The dysfunction of the epithelial barrier results in exceeding transport of the macromolecular substances to be absorbed into the deep tissue that has the potential to induce the antigen-related intestinal inflammation or hypersensitivity [4]. Two pathways are suggested in the antigen absorption under the epithelial barrier dysfunction. The common pathway is via the disintegrated tight junctions; another is via the transcellular transport [24], [25]. The present data indicate that the elevation of Cldn2 expression induces the drop of the TER in HT-29 and T84 monolayers, which mirrors that the paracellular pathway may be disintegrated; the luminal antigen may be transported to the deep tissue via the paracellular space, which needs to be further investigated. Meanwhile, the data also show that a plenty of antigens are bound by Cldn2 to form complexes; the latter can be internalized into the cytoplasm of the epithelial cells, which indicate the elevation of Cldn2 expression also contributes to the transcellular antigen transport. Previous studies observed that SEB could induce mononuclear cells to produce proinflammatory cytokine, interferon-γ, to compromise the intestinal epithelial barrier function [11], [26]. The present data further implicate that SEB can directly induce the gut epithelial barrier dysfunction by promoting the expression of Cldn2 in the epithelial cells.

The epithelial barrier dysfunction is a pathogenic feature in a number of diseases, such as food allergy [2], [4], asthma [3] and inflammatory bowel disease [6]. The present data indicate that the elevation of Cldn2 in epithelial cells plays an important role in the epithelial barrier dysfunction as well as in the pathogenesis of intestinal antigen-specific hypersensitivity. This is in line with our previous studies in which psychological stress-induced intestinal epithelial barrier dysfunction plays a critical role in the initiation of intestinal allergy [4]. Another finding of this study is that blocking Cldn2 can inhibit the induction of intestinal hypersensitivity. The fact implies that the Cldn2 has the potential to be a new therapeutic target in the disorders with Cldn2-overexpression-related epithelial barrier dysfunction.

CT can induce the intracellular cAMP production. Therefore upregulation of Cldn2 in colonocyte monolayers after prolonged incubation with CT may induce active chloride secretion, which has the potential to open the apical chloride channels to decrease in TER [8]. In line with previous reports, we also observed the drop of TER after the addition of CT to the culture of the epithelial monolayers. Furthermore, the data have shown another aspect of the action of CT on the epithelial cells. After exposure to CT, the expression of Cldn2 was increased; blocking Cldn2 abolished the CT-induced drop of TER. Since Cldn2 can form channels in the tight junction and increase in the ion secretion [8], the present data have enriched the understanding of CT-induced the gut epithelial barrier dysfunction, in which CT increases the expression of Cldn2, the latter further induces the ion secretion and decreases the TER.

## Supporting Information

Information S1 **Supplemental materials including Figures S1, S2, S3, S4, S5, S6 and Table S1.**
(DOCX)
