# Supplementary material for: Microbial Products Induce Claudin-2 to Compromise Gut Epithelial Barrier Function
Source: PLoS One. 2013 Aug 21;8(8):e68547. doi: 10.1371/journal.pone.0068547 (PMC3749177; doi:10.1371/journal.pone.0068547)
Supplement: Information S1 — Supplemental materials including Figures S1, S2, S3, S4, S5, S6 and Table S1. (DOCX) [file pone.0068547.s001.docx]

Supporting Information S1

**Claudin-2 induces gut epithelial barrier dysfunction**

**Running title**: Claudin-2 and gut epithelial barrier function

Xiaoyu Liu*^†^, Gui Yang^§†^, Xiao-Rui Geng^§†^, Yanjuan Cao*, Na Li*, Li Ma*, Si Chen*, Ping-Chang Yang*, Zhigang Liu*

*State Key Laboratory of Respiratory Disease for Allergy and Institute of Allergy & Immunology, Shenzhen University School of Medicine, Shenzhen, China. ^§^Longgang Central Hospital, ENT Hospital, Shenzhen ENT Institute, Shenzhen, China.

Correspondence to: Dr. Zhigang Liu, or Dr. Ping-Chang Yang. 3688 Nanhai Ave, Shenzhen, Guangdong 518060, China. [zhigangliu195910@126.com](mailto:zhigangliu195910@126.com) or pcy2356@163.com. Tel: 86 (0755) 86671907. Fax: 86 (0755) 86671906.

^†^Cao Y, Yang G and Geng XR share the first authorship

**Mice**

BALB/c mice (6-8 week old) were purchased from Charlse River Laboratories (Saint-Constant, QC, Canada). Mice were maintained in a specific pathogen free condition. The experimental procedures were approved by the Animal Care Committee at McMaster University.

**RNA isolation and quantitative real-time RT-PCR**

The total RNA was extracted from the cells using an RNeasy Mini kit. One micro gram RNA was reverse transcribed using the iScriptTMcDNA Synthesis Kit. The resulted complementary DNA was then subjected to qRT-PCR with SYBR Green by using a protocol provided by the manufacturer. One microliter of cDNA was amplified by using the RT-PCR primer sets of claudins as listed in Table 1. Relative quantification was determined with reference to β-actin RNA and analyzed using the comparative C_t_ method.

**Western blotting**

The small intestinal samples were homogenated and centrifuged at 15,000 rpm for 20 min at 4°C. The supernatant was collected; the levels of proteins were determined by Bio-Rad protein assay. A total of 50 µg protein were separated on SDS-PAGE gel and transferred onto nitrocellulose membrane. The membranes were then incubated with the primary antibodies (0.5-1 µg/ml) or isotype IgG (using as a negative control) overnight at 4°C. Reactions were developed using the Pierce ECL chemiluminescence substrate kit. The results were photographed with the UVP BioSpectrum Imaging System (Upland, CA, USA). Bands were semi-quantified by the densitometry. The density of the specific band was normalized to the intensity of β-actin band.

**ELISA**

Serum levels of cytokine and allergen-specific IgE were determined by ELISA. Blood samples were collected from each mouse. The sera were separated immediately and stored at -70°C until use. The levels of antigen specific IgE, IL-4, IL-5 and IL-13 in the sera or culture medium were measured by ELISA with commercial reagent kits following the manufacturer's instructions.

**Counting eosinophil, mononuclear cell and mast cell in the intestinal mucosa**

Numbers of eosinophils, mononuclear cells and mast cells in the small intestine were counted. Intestinal segments or biopsies were fixed with 4% paraformaldehyde (for mononuclear cells, eosinophil counting) or Carnoy solution (for mast cell counting) overnight and processed for paraffin sections. The sections were stained with hematoxylin (for mononuclear cells, eosinophil counting) or 0.5% toluidine blue (for mast cell counting) and observed under a light microscope.

**Carboxyfluorescein succinimidyl ester** (**CFSE) dilution assay**

T cell proliferation was assessed by the CFSE dilution assay. The lamina propria mononuclear cells (LPMC) were isolated by the gradient density centrifugation and labeled with CFSE. The LPMC were cultured in the presence of a specific antigen, HRP (20 ng/ml), for 4 days. The cells were collected, washed, stained with anti-IL-4, CD4 and CD3 antibodies, and analyzed by flow cytometry. The proliferated cell population was gated, the percentage of proliferated cells was calculated. Then, the percentage of CD3+ CD4+ IL-4+ T cells was further calculated regarding as proliferated Th2 cells.

**HT-29 and T84 cell culture**

HT-29 and T84 cells were purchased from ATCC (Manassas, VA, USA) as a passage of 30. Only passages 30–35 were used in the experiments. Cells were grown in the inserts of Transwells Dulbecco's modified eagle's medium supplemented with 10% heat inactivated fetal bovine serum, 1% nonessential amino acids, 1% glutamine, and 2% penicillin-streptomycin in 5% CO_2_ at 37°C and maintained. Upon 80–90% confluence, cells were split using 0.05% trypsin plus 1 mmol/L EDTA. Cells (10^6^ cells in 1 mL) were seeded on tissue culture transwell system (0.4 μm pore size, 1.2 cm^2^ surface area of the filter support) and grown for 5–7 days and used for experimentation. The media were changed every day. The polarized cells were used in this study.

**Gut epithelial monolayer transepithelial resistance (TER) measurements**

The TER was used as one of the indicators of epithelial barrier function that was measured with a Millicell-ERS apparatus (Millipore, Danvers, MA, USA). Experiments were carried out using the polarized epithelial cells with a TER ≥1000 Ω⋅cm^2^.

**Measurement of the permeability of epithelial monolayer**

Permeability of the epithelial barrier was used as one of the indicators of epithelial barrier function. Horseradish peroxidase (HRP; 20 ng/ml) was added to the apical chamber of transwells. Samples were obtained from the basal chambers at the time points of 12 h, 24 h, 36 h and 48 h. Samples were assayed for HRP by kinetic enzymatic assay. Briefly, the samples were added to phosphate buffer containing H_2_O_2_ and o-dianisidine. Enzyme activity was determined from the rate of increase in optical density at 460 nm. HRP transport was calculated as recovery of the original amounts of HRP.

**Lamina propria mononuclear cell (LPMC) isolation**

The small intestinal segments were collected and cut into about 2×2×2 mm and treated with predigestion solution (1× HBSS containing 5 mM EDTA and 1 mM DTT) at 37ºC for 30 min under slow rotation. After centrifugation (×1000 RPM, 10 min), the samples were incubated in the digestion solution (Dissolve 0.05 g of collagenase D, 0.05 g of DNase I and 0.3 g of dispase II in 100 ml of 1 × PBS) at 37ºC for 60 min under slow rotation. Cells were collected and filtered with a cell strainer. The LPMCs were isolated by gradient density centrifugation in percoll solution.

**Diagnosis of food allergy (following our established procedures):**

The recruitment of patients with food allergy and the healthy subjects were done at the Department of Gastroenterology, Zhengzhou University from Sep 2009 to Jun 2010.

*Case history*: The case history of food allergy was collected from each patient. Symptoms typically appear within minutes to two hours after the ingestion of the food to which he or she is allergic, which included a tingling sensation in the mouth, swelling of the tongue and the throat, difficulty breathing, hives, vomiting, abdominal cramps, diarrhea, drop in blood pressure, loss of consciousness, etc.

*Skin prick test*: One drop of extract of each fresh food (purchased from Beijing Union Hospital, Beijing, China) was applied to the patients' forearm. The skin covered by the drop of food antigen was pricked with 1 mm single-peak lancets. Positive control was performed using 10 mg/ml histamine dihydrochloride; the negative control using saline solution. The results were read after 15 min. The outline of each weal was documented using a piece of transparent paper to copy the wheal. The skin wheal areas were determined with scanned digital files using the software ImageJ. Skin prick tests were regarded positive when the area was larger than 7 mm^2^. Subjects with a weal area below 3 mm^2^ in positive controls were excluded from this study.

*Oral challenge with food antigens*: Oral challenges were performed in all the subjects in this study using one of the positive food allergens (revealed by skin test; usually chose the one had the largest wheal area). The food allergens in the skin prick test to screen the offending allergens in these patients are listed in Table S1 although some patients might also sensitize to other food antigens. The oral challenge was performed in a double blind manner. The antigen extracts or placebos were contained in coded capsules. The patients took 3 capsules of antigen or placebo; they were asked to take another 3 capsules (placebo or antigens) one week later. The study subjects were closely monitored for 4 hours following oral challenge in our department where treatment of anaphylaxis was readily available. The healthy controls were also oral challenged with a mixture of the antigens.

**Collection of biopsies from the duodenum of patients**

Six patients with food allergy and six patients with peptic ulcers but no food allergy were recruited to the present study at the Department of Gastroenterology of Zhengzhou University, the Second Hospital. The patients were undergone duodenal endoscopy to rule out the potential cancer in the upper digestive tract. Two pieces of biopsies were taken from each patient at non-diseased mucosa. The using human tissue in the study was approved by the human research ethic committee at Zhengzhou University. Informed written consent was obtained from each patient.

**RNA interference (RNAi) of Cldn2**

Following our previous experience (Jacob et al. J Biol Chem 2005;280(36):31936-48), HT-29 and T84 cells were cultured in transwells to 70-80% confluence. The Cldn2 shRNA or control shRNA was added to the insert of transwells following the manufacturer’s instruction. The gene of Cldn2 knockdown reached 90% on day 3 as shown by Western blotting; the effect lasted for at least two weeks (we only observed two weeks). The cells were used in experiments after day 3.

**Cldn2 gene overexpression**

pcDNA3-Cldn2 and pcDNA3-con (control) plasmids were a generous gift from Dr. Shaoheng He (Nanjing Medical University, China). Over-expression was achieved by transient transfection using JetPEI^™^ transfection reagent from Polyplus (Illkirch, France) following the manufactures protocol. After 48 h HT-29 cells were harvested to determine the levels of Cldn2 mRNA and protein. The cells were used for further experiments. The Cldn2 gene expression was presented in Fig.3D-E.

**Electron microscopy**

Following our reported procedures (Yang PC, et al. Enhanced intestinal transepithelial antigen transport in allergic rats is mediated by IgE and CD23 (FcepsilonRII). J Clin Invest. 2000;106:879-86), the HRP-treated HT-29 monolayer was processed for ultrathin section, stained with anti-HRP antibody, labeled with gold particle (10 nm) and observed with electron microscope.

**Statistical analysis**

The data were presented as mean ± SD. All statistical tests were two-sided Student's t-test between two groups or ANOVA in more than two groups; the statistical significance was defined as p less than 0.05.


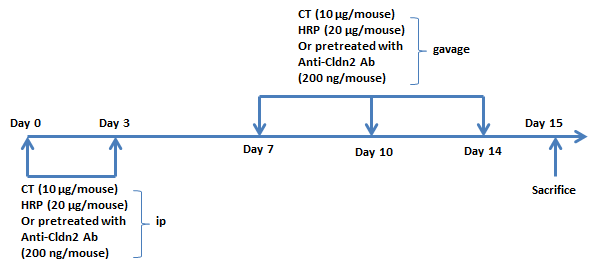


**Fig. S1. A protocol of intestinal sensitization to a specific allergen**. The grouped BALB/c mice (6 mice a group) were treated with saline (0.3 ml/mouse), or CT, or CT/HRP (in 0.3 ml saline; a group of mice was treated with CT/HRP and 200 ng/mouse anti-Cldn2 antibody as the group of horseradish peroxidase (HRP)/CTa) via i.p. on day 0 and day 3. The mice were treated with the same agents on day 7, day 10 and day 14 via gavage-feeding respectively. Mice were sacrificed on day 15. The blood and jejunal segments were sampled for further experiments (see below).


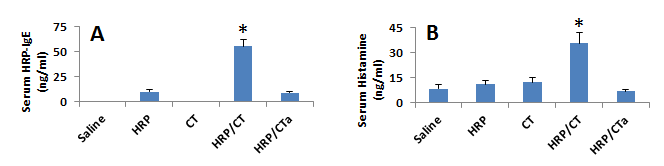


**Fig. S2. Levels of HRP-specific IgE and histamine in the sera**. The sera were collected from each mouse in figure S1 and analyzed by ELISA. The bars indicate the levels of HRP-specific IgE (A) and histamine (B). The data were presented as mean ± SD. *, p<0.01, compared with the group treated with HRP alone (A), or compared with the saline group (B). N=6.


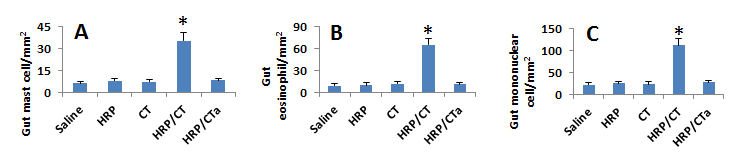


**Fig. S3. Frequencies of cellular components in the jejunum**. The jejunal segments were excised from each mouse (see figure S1) at sacrifice, and fixed with Carnoy solution (A) or paraformaldehyde (B and C). The tissues were then processed for paraffin sections. The sections were stained with 0.5% toluidine blue (A) or eosin & hematoxylin (B and C). The slides were coded; the observers were not aware of the code. Under a light microscope, 20 fields (×200) were randomly selected. The number of mast cells, eosinophils and mononuclear cells were counted. The bars indicate the summarized cell number of mast cells (A), eosinophils (B) and mononuclear cells (C). The data were presented as mean ± SD. *, p<0.01, compared with the saline group.


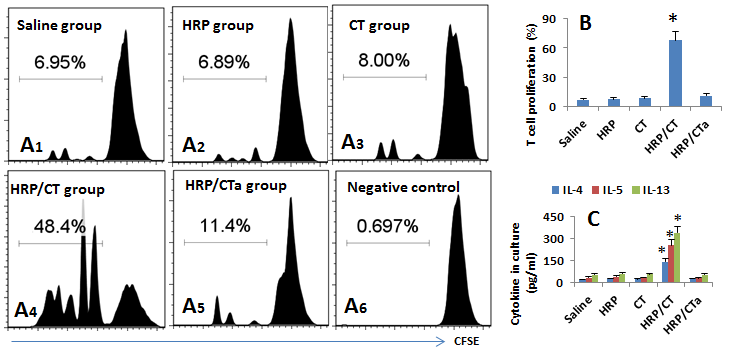


**Fig. S4. Antigen-specific Th2 response in the small intestine**. The small intestinal segments were obtained from each mouse (see figure S1). The lamina propria mononuclear cells were isolated, CD3+ CD4+ CD25- T cells were purified with magnetic cell sorting and labeled with CFSE and cultured in the presence of specific antigen HRP for 4 days; the cells were collected and stained with antibodies of CD3, CD4 and IL-4. The cells were then analyzed by flow cytometry. The histograms (A) indicate the frequency of the proliferated T cells. The bars in B indicate the summarized frequency of proliferated cells in A. The gated cell population in HRP/CT group was further analyzed by the gating technique; the results showed that the rate of CD3+ CD4+ IL-4+ T cells in the gated cells was 93.3 ± 14.5%. The data indicate most the proliferated cells are Th2 cells. C, the culture supernatants were collected at the end of culture and analyzed by ELISA. The bars indicate the levels of IL-4, IL-5 and IL-13 in the culture medium. N=6 of each group. CTa: Mice were i.p. injected with ant-CT antibody (100 ng/mouse) 30 min prior to each exposure to the specific antigen, HRP.


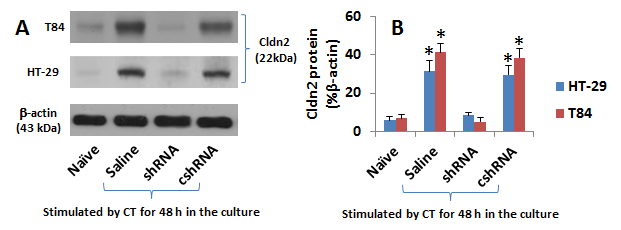


**Fig. S5. Knockdown of Cldn2 gene.** A, the immune blots indicate the contents of Cldn2 in T84 or HT-29 cells. B, the bars indicate the summarized integrated density of the immune blots of A. The data in the bar graph were expressed as mean ± SD from 3 separate experiments. *, p<0.01, compared with naïve cells. shRNA: Cldn2 shRNA. cshRNA: Control shRNA. Naïve: Naïve HT-29 or T84 cells.


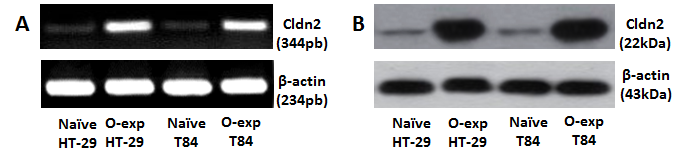


**Fig. S6. Over expression of Cldn2**. A, the PCR products indicate the expression of Cldn2 mRNA in HT-29 cells. B, the immune blots indicate the Cldn2 protein levels in HT-29 cells. O-exp: Overexpression of Cldn2. The data represent 6 separate experiments.

Table S1. Demographic data of patients (pt)

| Pt# | Age | Sex | Food allergens (determined by skin test) | Other  allergies | Specific IgE (ng/ml) | Total IgE (ng/ml) |
| --- | --- | --- | --- | --- | --- | --- |
| Patients with food allergy | | | | | | |
| 1 | 34 | M | Milk, fish, egg | Asthma | 61.2 | 94.5 |
| 2 | 28 | F | Egg, milk | Asthma | 74.5 | 88.6 |
| 3 | 36 | M | Soy bean, wheat |  | 57.3 | 104.5 |
| 4 | 51 | F | Milk, fish, wheat |  | 41.5 | 114.2 |
| 5 | 38 | F | Peanut, soy bean, sesame | Rhinitis | 38.5 | 97.5 |
| 6 | 29 | M | See food, peanut, milk |  | 45.6 | 51.9 |
|  | | | | | | |
| Patients with peptic ulcer, no allergy | | | | | | |
| 1 | 33 | M |  |  |  | 15.6 |
| 2 | 42 | F |  |  |  | 14.3 |
| 3 | 38 | M |  |  |  | 21.4 |
| 4 | 54 | M |  |  |  | 16.2 |
| 5 | 39 | F |  |  |  | 14.2 |
| 6 | 48 | F |  |  |  | 21.2 |
